# The research status and prospects of nanomaterials in wound healing: A scientometric study

**DOI:** 10.1097/MD.0000000000037462

**Published:** 2024-03-15

**Authors:** Songxia Xia, Renxian Wang, Xueshan Bai, Jing-Jun Nie, Dafu Chen, Li Teng, Liya Yang

**Affiliations:** a Plastic Surgery Hospital, Chinese Academy of Medical Sciences and Peking Union Medical College, Beijing, China; b Laboratory of Bone Tissue Engineering, Beijing Laboratory of Biomedical Materials, National Center for Orthopaedics, Beijing Research Institute of Traumatology and Orthopaedics, Beijing Jishuitan Hospital, Capital Medical University, Beijing, China; c JST sarcopenia Research Centre, National Center for Orthopaedics, Beijing Research Institute of Traumatology and Orthopaedics, Beijing Jishuitan Hospital, Capital Medical University, Beijing, China.

**Keywords:** bibliometric analysis, nanomaterial, scientometric study, visualization, wound healing

## Abstract

Nanotechnology and nanomaterials have swiftly influenced wound healing, propelling the development of wound-healing nanomaterials. Therefore, it’s crucial to gather essential information about prominent researches in this domain. Moreover, identifying primary directions and related frontiers in wound healing and nanomaterials is paramount. This will enhance our comprehension of the current research landscape and foster progress in this field. Retrieved from the Web of Science core database, a total of 838 relevant studies published from 2013 to 2022 were analyzed through bibliometric visualization tools such as CiteSpace, VOSviewer, and Bibliometrics Online Analysis Platform. The annual study count has been rising steadily, primary contributors to this field include China, India, and the United States. The author with the highest output is Zangeneh, Akram, while Grumezescu, Alexandru Mihai garners the most citations. Chinese Academy of Sciences emerges as the leading institution, with *Nanomaterials* as the predominant journal. The keyword “antibacterial” signals prevailing and forthcoming trends in this domain. This study presents the first scientometric study and bibliometric visualization for wound healing-related nanomaterials, shedding light on research hotspots and trends. Over the course of the decade from 2013 to 2022, enthusiasm for nanomaterials in wound healing research has surged, auguring well for upcoming investigations.

## 1. Introduction

The skin, being the body’s largest organ, acts as the primary protective barrier against external environmental threats. Its functions include protection, secretion, excretion, sensation, and immunity.^[1]^

Trauma is the consequence of various harmful internal and external stimuli that disrupt the structural and functional integrity of the skin. Burn injuries, physical injuries, surgical incisions, and diabetes are among the most frequent causes of wound formation.^[2]^ Following skin integrity compromise, the human body is at risk of encountering microbial invasion and experiencing fluid loss. The human body has an innate capacity to swiftly activate wound healing mechanisms, aiming to restore the integrity of the skin.^[3]^ Nevertheless, under specific conditions like uncontrolled inflammation, vascular/neurological impairment and wound infection, wound healing might experience delays and lead to chronic wound formation.^[4]^ Projections indicate that the global annual expenditure on chronic wound care products is expected to surpass 22 billion dollars by the year 2024.^[5]^

According to conventional classification based on healing time frames, skin wounds are broadly classified into 2 categories: acute and chronic.^[6]^ Acute wounds typically follow a predictable and sequential process of tissue repair, resulting in the prompt restoration of skin integrity in a matter of weeks or within a month. Conversely, for chronic wounds, the barrier defect lacks a repair pattern that follows a sequential and time-dependent sequence, leading to impaired healing or deterioration.^[7]^ Based on the etiology, chronic or non-healing wounds are primarily categorized as wounds related to diabetes, vascular disorders, and local pressure sources, although wound-specific factors such as inflammation, infection, radiation, and other complications may also contribute. Numerous systemic factors, such as immune response, nutrition, aging, psychological stress, and other comorbidities, may lead to delayed wound healing.^[8,9]^ Furthermore, wounds of skin can be categorized based on depth: superficial wounds (limited to the epidermis), partial-thickness wounds (damage to the deep layers of the epidermis and dermis), and full-thickness wounds (damage to the entire skin, including subcutaneous fat and deep tissues).^[7]^

There remain numerous challenges in achieving rapid and high-quality promotion of skin wound healing. Recently, the application of nanomedicine has led to significant progress in the wound healing nanomaterials. This has resulted in the increasing utilization of nanomaterials for promoting the healing of various types of wounds.^[10]^

A wide range of nanomaterials, such as polymeric nanomaterials, inorganic nanomaterials and extracellular vesicles-based nanomaterials, have exhibited beneficial properties in promoting wound healing, showcasing their potential therapeutic effects.^[11]^ Nanomaterials possess distinctive physical and chemical properties and functionalities attributed to the quantum size effect and surface effect.^[12]^ Certain nanomaterials exhibit antibacterial, pro-angiogenic and antioxidative properties, enabling them to enhance wound healing by regulating the microenvironment, managing infections, and stimulating angiogenesis and re-epithelialization.^[13]^ Additionally, because of their considerable specific surface area, nanomaterials can function as carriers to deliver therapeutic drugs, effectively modulating wound healing processes, provide antibacterial agents and growth factors, thereby combating infections and stimulating burn wound healing.^[14]^

Generally, nanomaterials are defined as materials with dimensions ranging from 1 to 1000 nm in at least one direction, but they are typically characterized by a diameter falling within the 1 to 100 nm range.^[15]^ Nevertheless, there is no universally agreed-upon definition for nanomaterials at the international level, and different organizations have different opinions on how to define them.^[16]^ Based on nanomaterials’ crystalline structures and chemical compositions, Gleiter^[17]^ have classified nanomaterials into different categories. The majority of current nanoparticles and nanostructured materials can be divided into 4 material-based categories: carbon-based nanomaterials; inorganic-based nanomaterials; organic-based nanomaterials; composite-based nanomaterials.^[18]^ However, the dimensionality of nanomaterials was not taken into account. Pokropivny and Skorokhod^[19]^ proposed a novel classification system for nanomaterials, incorporating the recently developed composite materials like 0D, 1D, 2D, and 3D nanomaterials. This system takes into account the dimensionality of nanomaterials, and the electron mobility along these dimensions plays a crucial role in determining its characteristics.^[15]^

Nanomaterials have strong prospects for research and wide clinical applications within the context of wound healing and regeneration. Nanomaterials play a crucial role in both acute and chronic wound healing. Healthcare professionals, pharmaceutical experts, and biomedical researchers should pay increased attention to the application of nanomaterial formulations in wound healing treatments to contribute to the progressing field of medical wound therapy.^[20,21]^ A large number of publications have been released on wound healing and regeneration using nanomaterials both domestically and abroad. The study of nanomaterials in wound healing is interdisciplinary, requiring scientists from engineering and medical fields to have a grasp of advancements in each other’s disciplines for effective collaboration. Scientometric study provides a rapid and comprehensive understanding of the evolution of knowledge and academic impact in multidisciplinary collaborative research.^[22]^ It is crucial for exploring their contributions to overall scientific progress, but there has yet to be a systematic review of these articles using bibliometric analysis to evaluate the distribution of researchers, affiliations, countries, as well as the trends, focuses, and frontiers of this research domain. The objective of this study is to offer a visual summary of the current research concerning the use of nanomaterials in wound healing, while providing direction and ideas for future research. To achieve this, Web of Science database was searched for articles on the forefront, hotspots, and trends of nanomaterials and their applications in wound healing, and we conducted data mining and analysis using VOSviewer and CiteSpace scientometric software based on Social Sciences Citation Index literature.

## 2. Materials and methods

### 2.1. Search strategy and selection method

Web of Science is a valuable academic database that covers natural sciences, social sciences, and humanities. It indexes over 21,000 scholarly journals and includes conference proceedings from various academic conferences. The database is comprised of important components, including Science Citation Index-Expanded, Social Sciences Citation Index, Conference Proceedings Citation Index, and Arts & Humanities Citation Index. Web of Science offers reference tracing and citation reporting tools, facilitating the analysis of citation source characteristics within the literature. This enables researchers to identify hotspots and trends in specific fields of research.

Searching the Medical Subject Headings (MeSH) database (https://www.ncbi.nlm.nih.gov/mesh) for relevant terms “nanomaterial” and “wound healing” yielded a number of alternative terms. All identified terms were searched on Web of Science to ensure comprehensive coverage of related literatures.

The study relied on publicly available data and previously published articles. Ethical approval was not required since the articles did not include any personally identifiable information about the subjects.

**Figure 1.**
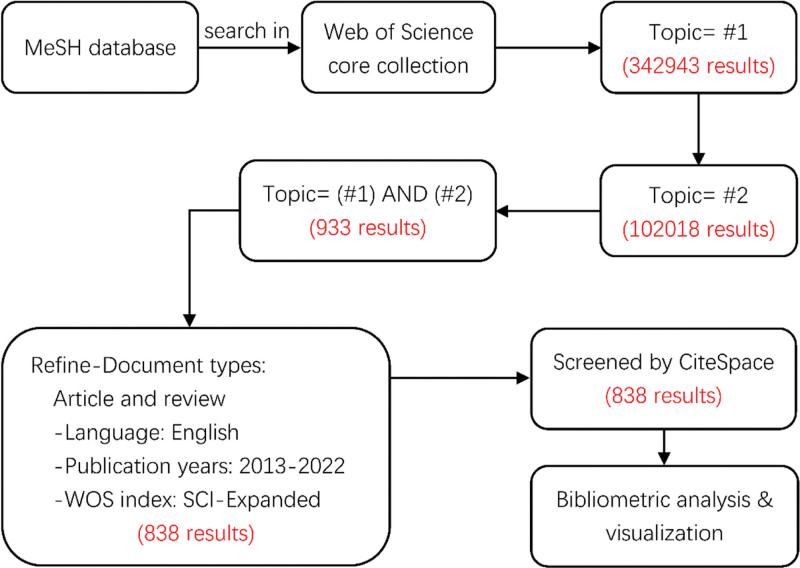
Flow chart about the search strategy.

Figure 1 shows the search strategy.

#1: TS=(Nanostructures) OR TS=(Nanostructure) OR TS=(Nanostructured Materials) OR TS=(Material, Nanostructured) OR TS=(Materials, Nanostructured) OR TS=(Materials, Nanostructured) OR TS=(Nanomaterials) OR TS=(Nanomaterial)

#2: TS=(Wound Healing) OR TS=(Healing, Wound) OR TS=(Healings, Wound) OR TS=(Wound Healings) OR TS=(Re-Epithelialization) OR TS=(Re Epithelialization) OR TS=(Wound Epithelialization) OR TS=(Epithelialization, Wound)

#3: TS=(#1) AND (#2) AND DOCUMENT TYPES: (ARTICLE OR REVIEW) AND LANGUAGES: (ENGLISH) AND WEB OF SCIENCE INDEX: (Web of Science Core Collection. SCI), with the timespan of 2013 to 2022.

Through the search strategy outlined above, after eliminating duplicate records with unclear publication years, we retrieved 838 research studies from the Web of Science in total for further visual analysis. The search process was concluded on March 26th, 2023.

### 2.2. Methodology

Analysis of the search results for nanomaterials in the field of wound healing, including publication year, country or region, affiliation, journal, core authors, keywords, and distribution of key reference literature. CiteSpace (version 6.2.R2; https://citespace.podia.com), VOSviewer (version 1.6.19; https://www.vosviewer.com), Bibliometric Online Analysis Platform (https://bibliometric.com), and Microsoft Excel 2019 were used for visual analysis of the literature metrics of nanomaterials.

CiteSpace and VOSviewer are both scientometric visualization tools. CiteSpace can illustrate collaborative connections among research entities by visualizing networks and centrality metrics for countries, authors, and institutions. Additionally, it can unveil the knowledge foundation and emerging trends in a research field through co-citation analysis.^[23]^ This study employed the logarithmic likelihood ratio method for CiteSpace analysis. Each node’s circumference reflects the number of literatures that meet the criteria, while the proportion in the outermost circle represents centrality, indicating the node’s ability to connect to others. Sigma evaluates a node’s role in citation activity based on centrality and burstiness. The line thickness between nodes represents their association strength. Additionally, VOSviewer can visualize the scientific landscape using Linlog/modularity methods based on network, coverage and density patterns.^[24]^ Each node’s weight is based on citation or document count, while the color represents the publication year on average or cluster type which depends on the chosen analysis mode. The Bibliometric Online Analysis Platform facilitates network cooperation among countries/regions. Microsoft Excel 2019 serves as a fundamental tool for importing, sorting data, and creating tables.

## 3. Results

There were 838 eligible documents between 2013 and 2022, containing 610 (72.79%) articles and 228 (27.21%) reviews.

**Figure 2.**
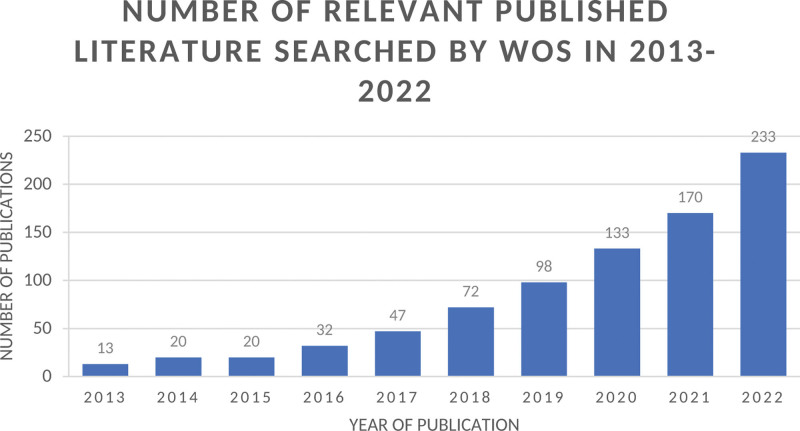
Distribution of publications on nanomaterial in wound-healing according to the year.

Based on the information depicted in the statistical figure (Fig. 2), only 13 articles related to nanomaterials in wound healing were released in 2013, and the quantity of relevant studies has been steadily rising since that time. In 2022, there were 233 published articles. The growth rate of publications from 2017 to 2022 has shown a significant acceleration, and a continuous rise in the number of relevant studies over the years from 2013 to 2022 can be indicated. This surge in interest highlights the increasing attention nanomaterials have garnered in the wound healing domain.

### 3.1. Countries and regions

The pertinent literature was released by 65 countries and regions from 2013 to 2022.

According to Table 1, the top 10 countries and regions with the highest number of related publications include China, India, the United States, Iran, South Korea, Spain, Italy, Saudi Arabia, the United Kingdom, and Brazil, respectively. Among them, China has published the most papers (312, accounting for 37.23%), followed by India (130, 15.51%), and the United States (126, 15.04%).

**Table 1:** The countries and regions with the top 10 number of published literatures in 2013 to 2022.

No.	Country	Documents	Citations
1	People R China	312	10,671
2	India	130	3216
3	USA	126	4851
4	Iran	79	2650
5	South Korea	41	1131
6	Spain	33	1222
7	Italy	32	701
8	Saudi Arabia	32	905
9	England	29	1212
10	Brazil	23	907

**Figure 3.**
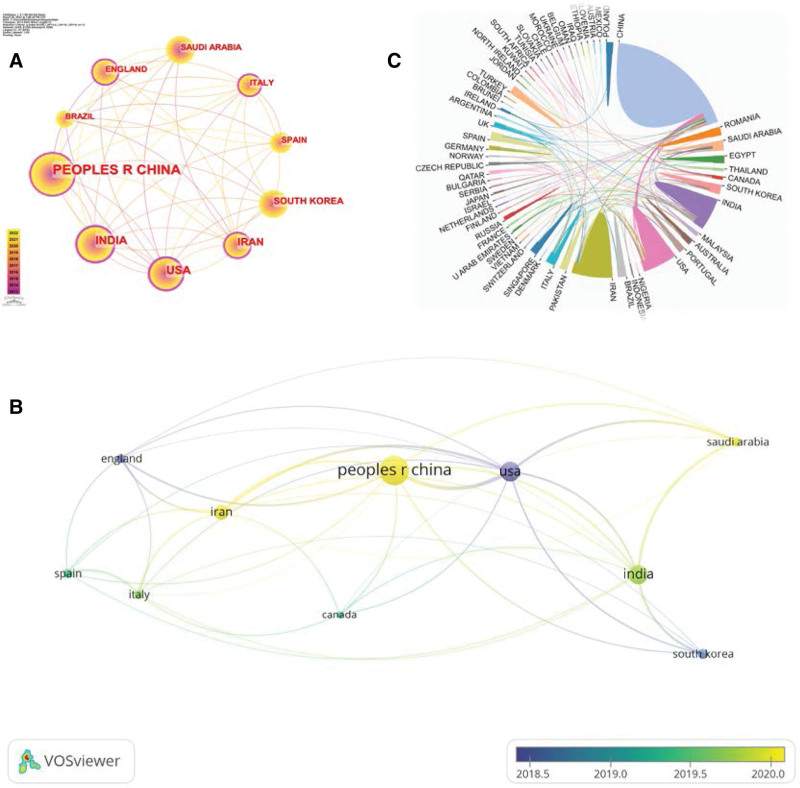
Visualization of country/region collaboration analysis. (A) Collaboration analysis of the top 10 countries/regions with the highest number of publications. The node size represents the number of related publications of that country. Nodes denoted by a purple circle imply that the country’s intermediary centrality is equal to or greater than 0.1. (B) Superimposed visualization of the main country/region co-authorship relationships. The size of each node represents the number of publications of that country. The thickness of the lines connecting the nodes reflects the level of collaboration between nations. The color of each node corresponds to the main publication time of that country. (C) Scientific cooperation of nanomaterial-related countries/regions in the field of wound-healing. The size of each color block between countries represents the number of publications of that country. The connections between nations indicate partner relationships.

Figure 3A illustrates the collaboration among these top 10 countries, with nodes surrounded by purple circles indicating those with a betweenness centrality score above 0.1. Betweenness centrality quantifies a node’s capacity to function as an intermediary between 2 other nodes. A value above 0.1 signifies a critical hub that connects others. Out of these countries, those having a betweenness centrality score above 0.1 are China, India, the United States, Iran, Italy, and the United Kingdom. The publications authored by Chinese researchers were cited more frequently than those of other countries, with a total citation count of 10,671.

In accordance with the number of publications, the nations with the closest collaboration to China are the United States, Iran, and India. According to the time overlay graph, the collaboration among major countries was predominantly focused in 2020, with the United States, South Korea, and the United Kingdom exhibiting a higher concentration of publications in the early period, while China, Iran, and Saudi Arabia had relatively concentrated publication time in the later period (Fig. 3B and C).

Based on the above data, China dominates the research of nanomaterials in wound healing field.

### 3.2. Journals

Based on the VOSviewer analysis, 279 journals released related papers from 2013 to 2022. After merging different abbreviations of the same journal, 48 journals met the threshold setting (The threshold criteria were set as follows: a minimum of 5 articles for the source and a minimum citation count of 0.) Information on journal impact factor (JIF) and journal citation indicator (JCI) was obtained from Journal Citation Reports, Clarivate (https://jcr.clarivate.com/jcr/home).

JIF is a metric used to evaluate the citation rate of articles published in journals. Given the diverse reference patterns across topics, JIF lacks standardization for this variation. Therefore, it is advisable to utilize JCI for comparing journals within the same classification. JCI stands for the average category-normalized citation impact of citable literature published in journals during the last 3 years. Within the same category, JCI averages to 1. Journals which have a JCI of 1.5 are regarded as having 50% higher citation impact than their counterparts. (https://jcr.clarivate.com/). The journal’s JIF and JCI were obtained from the 2021 data, which was updated in June 2022.

During the period from 2013 to 2022, the ranking of journals with the most publications of relevant literature is listed in Table 2. The top 3 journals with the highest number of publications are *Nanomaterials, ACS Applied Materials & Interfaces*, and *Acta Biomaterialia*. The JIF of the journals ranged from 4.036 to 16.744, with an average JIF of 8.919 for each journal. Among them, 3 journals have JCI values greater than 1.5, namely *Carbohydrate Polymers, Chemical Engineering Journal*, and *Acta Biomaterialia*. On average, these journals demonstrate a higher citation impact compared to others within their respective categories.

**Table 2:** Top 10 journals in the number of relevant literatures published in 2013 to 2022.

No.	Journal	2021		Documents	Citations
		JIF	JCI		
1	*Nanomaterials*	5.719	0.79	29	1254
2	*ACS Applied Materials & Interfaces*	10.383	1.48	27	940
3	*Acta Biomaterialia*	10.633	1.72	18	970
4	*Biomaterials Science*	7.59	1.14	17	422
5	*International Journal of Nanomedicine*	7.033	1.18	15	456
6	*Nanoscale*	8.307	1.25	15	859
7	*Chemical Engineering Journal*	16.744	2.13	14	542
8	*International Journal of Biological Macromolecules*	8.025	1.42	14	659
9	*RSC Advances*	4.036	0.57	14	458
10	*Carbohydrate Polymers*	10.723	2.19	13	1171

JCI = Journal Citation Indicator, JIF = journal impact factor.

According to the total citation count, 11 journals have been cited more than 500 times (Table 3), listed in descending order. The 3 leading journals with the highest citation count are *ACS Nano, Nanomaterials*, and *Carbohydrate Polymers*. Based on the JIF and JCI metrics, the top-ranked journals are *ACS Nano* (JIF = 18.027, JCI = 2.51), *Chemical Engineering Journal* (JIF = 16.744, JCI = 2.13), and *Small* (JIF = 15.153, JCI = 1.9). These journals are authoritative in the research of nanomaterials in the field of wound healing.

**Table 3:** Journals with relevant literatures cited more than 500 in 2013 to 2022.

No.	Journal	2021		Documents	Citations
		JIF	JCI		
1	*ACS Nano*	18.027	2.51	12	1616
2	*Nanomaterials*	5.719	0.79	29	1254
3	*Carbohydrate Polymers*	10.723	2.19	13	1171
4	*Acta Biomaterialia*	10.633	1.72	18	970
5	*ACS Applied Materials & Interfaces*	10.383	1.48	27	940
6	*Nanoscale*	8.307	1.25	15	859
7	*Materials Science & Engineering C-Materials for Biological Applications*	8.457	1.38	9	695
8	*International Journal of Biological Macromolecules*	8.025	1.42	14	659
9	*Materials*	3.748	0.62	12	547
10	*Chemical Engineering Journal*	16.744	2.13	14	542
11	*Small*	15.153	1.9	12	521

JCI = Journal Citation Indicator, JIF = journal impact factor.

In summary, taking into account the quantity of published literature, citation rates, as well as JIF and JCI values, *Nanomaterials* and *Acta Biomaterialia* emerge as more dynamic and impactful sources on the subject of nanomaterials in wound healing.

### 3.3. Affiliations

During the period of 2013 to 2022, the institutions with the highest number of publications on the topic are listed in Table 4. Except for the 10th ranked institution from India, all others are from China and Iran. The Chinese Academy of Sciences (CAS) has the highest number of publications (46, 5.49%), followed by Sichuan University (21, 2.51%) and Shanghai Jiao Tong University (19, 2.27%).

**Table 4:** Top 10 affiliations in the number of relevant literatures published in 2013 to 2022.

No.	Institution	Documents	Citations	Link	Total link strength
1	Chinese Academy of Sciences	46	3228	261	489
2	Sichuan University	21	519	77	103
3	Shanghai Jiao Tong University	19	393	69	95
4	Islamic Azad University	18	652	153	180
5	University of Chinese Academy of Sciences	18	713	73	100
6	Razi University	15	706	84	215
7	Ilam University of Medical Sciences	13	514	64	191
8	University of Tehran	13	495	113	173
9	Iran University of Medical Sciences	12	614	134	164
10	SRM Institute of Science & Technology	12	177	52	82

Among the top 10 affiliations with the most published literatures, 3 have received more than 700 citations. Among all affiliations that have published relevant literature, 7 have been cited over 700 times. Chinese Academy of Sciences is far ahead of the second-ranked institution with regard to the number of studies published and citations. Interestingly, although the number of studies published by Northwestern Polytechnical University is less than 25% of University of the Chinese Academy of Sciences, its citation count is even higher than that of University of the Chinese Academy of Sciences (Tables 4 and 5).

**Table 5:** Top 10 affiliations in the number of relevant literatures cited in 2013 to 2022.

No.	Institution	Documents	Citations	Link	Total link strength
1	Chinese Academy of Sciences	46	3228	261	489
2	Politehnica University of Bucharest	10	998	155	196
3	Tianjin University	8	755	86	113
4	Northwestern Polytechnical University	4	717	111	147
5	University of the Chinese Academy of Sciences	18	713	73	100
6	Xi’an Jiaotong University	9	709	117	185
7	Razi University	15	706	84	215
8	University of Hong Kong	5	670	68	89
9	Hubei University	4	669	68	89
10	National Center for Nanoscience and Technology, China	2	658	80	109

**Figure 4.**
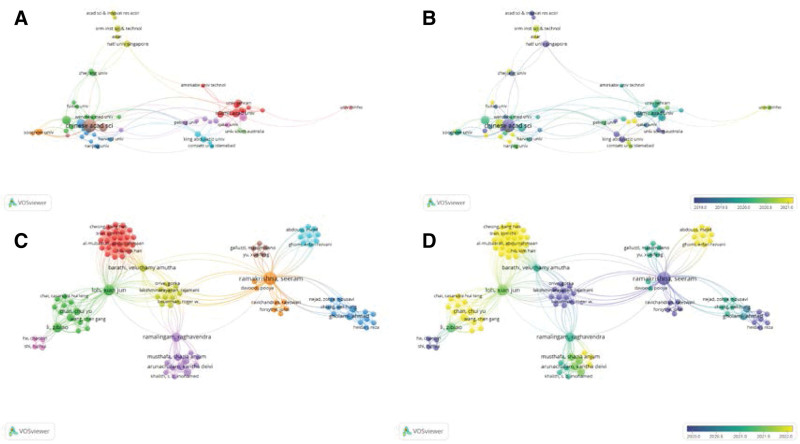
Visualization of affiliation and co-authorship analysis. (A) Network visualization of coauthors based on main affiliations. Nodes sharing the same color are part of the same cluster. (B) Overlay visualization of coauthors based on main affiliations. Node colors correspond to the main publication time of the respective affiliation or author’s literature. (C) Network visualization of coauthors based on main authorship. Nodes sharing the same color are part of the same cluster. (D) Overlay visualization of coauthors based on main authorship. Every node presents an affiliation or author. Node colors correspond to the main publication time of the respective affiliation or author’s literature. Node size indicates the number of publications. The thickness of connections between nodes indicates the strength of affiliation or collaboration between authors.

During the period of 2013 to 2022, Chinese Academy of Sciences had the highest number of links (261) and total link strength (489) among all affiliations, indicating the most frequent and close relationships between CAS and other organizations. CAS had established partnerships with affiliations like University of Chinese Academy of Sciences, Shanghai Jiao Tong University, National University of Singapore, and University of California, Los Angeles. Collaboration between affiliations mainly occurred after 2019 (Fig. 4A and B).

### 3.4. Authors

After consolidating various author name abbreviations, the VOSviewer analysis showed 4642 authors in total who contributed to related literature between 2013 and 2022. Among them, 16 authors met the threshold settings. (The threshold criteria for authors were set as follows: a minimum of 5 publications and 0 citations.)

Out of the 11 authors whose publications have been cited over 400 times, 7 are from China, 3 are from Romania, and 1 is from Iran. Zangeneh, Akram from Razi University is the most prolific author with 12 published papers. Grumezescu, Alexandru Mihai from Politehnica University of Bucharest is the most cited author with 792 citations, followed by Yin, Wenyan from the Chinese Academy of Sciences with 741 citations. The H-index is a metric employed to assess both the quantity and impact of academic production.^[25]^ Among the authors, the top 4 individuals possess the highest H-index values are Liu, Xiangmei (67), Guo, Baolin (66), Gu, Zhanjun (65), and Wu, Shuilin (65). Most authors are affiliated with multiple institutions, which undoubtedly directly promote collaboration between institutions, and these institutions have high publication or citation frequencies (Tables 4, 5, and 6).

**Table 6:** A list of authors with more than 400 citations from 2013 to 2022.

No.	Author	Affiliation	Citations	Documents	Total link strength	H-index
1	Grumezescu, Alexandru Mihai	Politehnica University of Bucharest	792	5	175	42
2	Yin, Wenyan	Chinese Academy of Sciences	741	3	316	54
3	Gu, Zhanjun	Chinese Academy of Sciences	740	2	311	65
4	Zhao, Yuliang	Chinese Academy of Sciences	740	2	311	46
5	Wu, Shuilin	Tianjin University/Hubei University	669	4	216	65
6	Liu, Xiangmei	Hubei University	669	4	216	67
7	Ficai, Anton	Politehnica University of Bucharest	646	3	76	34
8	Wang, Xianbao	Hubei University	613	3	190	60
9	Guo, Baolin	Xi’an Jiaotong University	577	4	230	66
10	Andronescu, Ecaterina	Politehnica University of Bucharest	577	2	53	34
11	Zangeneh, Akram	Razi University	465	12	285	25

The partnership among authors is dispersed. The largest collaborative network among authors who published related papers is revealed in Figure 4C and D, which includes 103 individuals, and the majority of their collaborations took place after 2020.

### 3.5. Keywords

**Figure 5.**
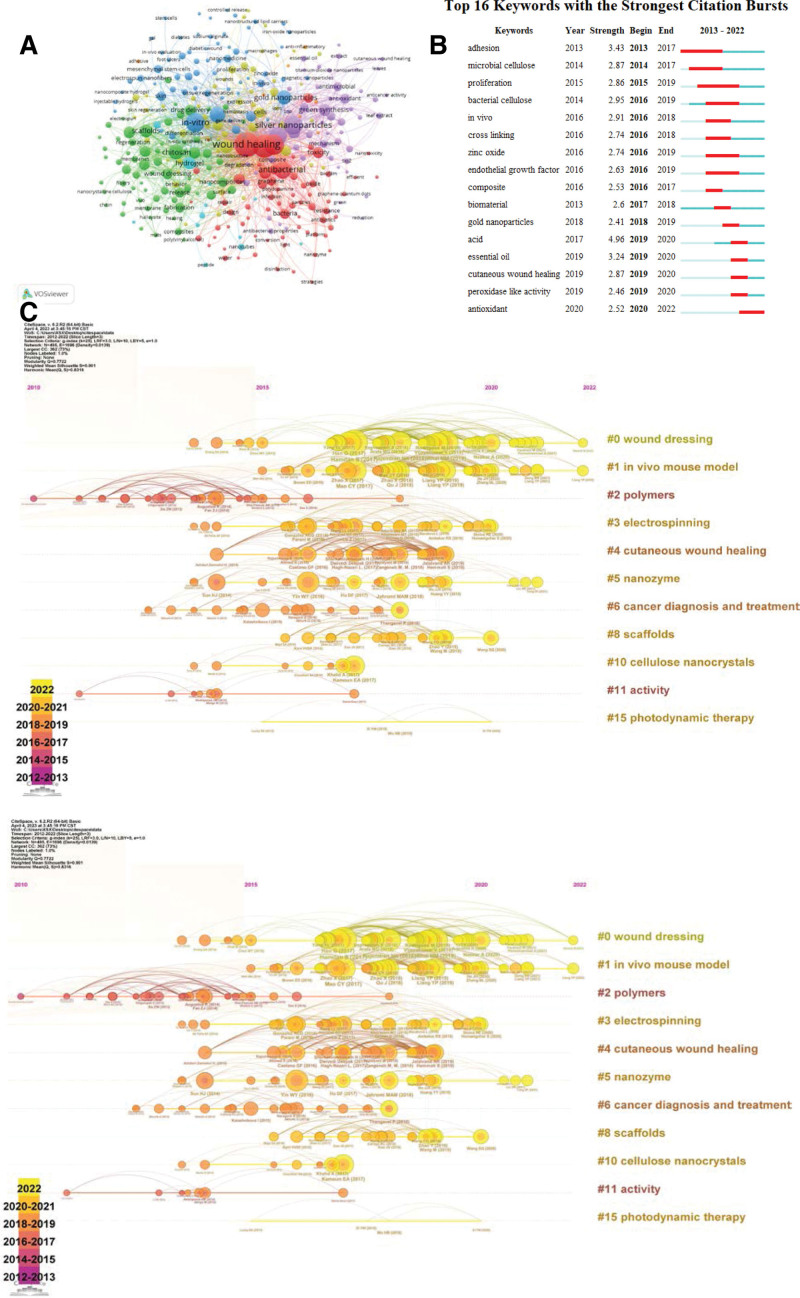
Research hotspots and frontiers. (A) Network visualization of co-occurring keywords. Every node corresponds to a distinct keyword, with the size indicating the frequency of occurrence. The thickness of the links between nodes represents the strength of the co-occurrence relationship. Nodes sharing the same color are part of the same cluster. (B) The top 16 keywords with the highest citation frequency. The most pronounced citation burst indicates a rapid increase in the frequency of occurrence within a brief timeframe. The red bars signify the duration of the keyword burst. (C) Timeline of co-cited references. Every node presents a literature, with the y-axis indicating the cluster that the node belongs to and the x-axis indicating the publication time. The links between nodes indicate the relationship between literatures.

After combining different forms of writing (abbreviations, hyphens, etc.) for the same keyword into a single entry, VOSviewer analysis revealed 4007 keywords during the period of 2013 to 2022, of which 326 met the threshold setting (minimum keyword occurrence of 5 times). Apart from subject-related keywords such as “wound healing,” “nanoparticles,” “nanomaterials,” and status-related vocabulary such as “in vitro,” the main keywords with high frequency of occurrence include “silver,” “antibacterial,” and “drug-delivery” (Fig. 5A). These keywords are broadly classified into 4 categories: inorganic material, organic material, material technic, and medical focus, as shown in Table 7. The most frequently occurring inorganic materials during 2013 to 2022 were silver, gold, and graphene oxide, while the most frequently occurring organic materials were hydrogel, chitosan, and hyaluronic acid. The most frequently occurring material technics during 2013 to 2022 were scaffolds, green synthesis, and tissue engineering. The most frequently occurring medical focus during 2013 to 2022 were antibacterial, drug delivery, and toxicity.

**Table 7:** Co-occurrence statistics of major keywords during 2013 to 2022.

No.	Inorganic material	Occurrence	Organic material	Occurrence	Material technic	Occurrence	Medical focus	Occurrence
1	silver	178	hydrogel	111	scaffolds	66	antibacterial	241
2	gold	76	chitosan	90	green synthesis	56	durg delivery	147
3	graphene oxide	47	hyaluronic acid	20	tissue engineering	51	toxicity	89
4	graphene	31	bacterial cellulose	23	electrospinning	47	delivery	70
5	zno	28	endothelial growth factor	18	scaffold	24	antimicrobial	53
6	carbon nanotubes	28	leaf extract	17	films	22	tissue engineering	51
7	oxide	20	polymer	17	electrospun nanofibers	18	wound dressing	46
8	zinc-oxide	18	essential oil	14	cross-linking	18	angiogenesis	43

The most explosive keywords can indicate sudden changes in the research focus within a certain period, reflecting the hotspots and frontiers in a specific area of research. Figure 5B shows the 16 most cited keywords from 2013 to 2022. The most prominent word is “adhesion,” with a strength of 3.43 from 2013 to the end of 2017. Keywords such as “adhesion,” “proliferation,” and “endothelial growth factor” indicate the focus of research on nanomaterials at the cellular level, while keywords such as “zinc oxide” and “gold nanoparticles” indicate the hotspots in material category research. The term “cutaneous wound healing” indicates the focus and frontier of nanomaterial research in the field of wound healing, focusing on skin wounds, while research on “peroxidase-like activity” and “antioxidant” initiated in 2019, this area of research continues to be a focal point.

### 3.6. Citations

Table 8 showcases the 10 most cited literature. This approach aids in gathering authoritative articles within a certain field and identifying the emergence of past concepts at specific time points. Clearly, the article with the highest citation frequency is “*Functionalized nano-MoS*_*2*_ *with peroxidase catalytic and near-infrared photothermal activities for safe and synergetic wound antibacterial applications*,” authored by Yin, Wenyan, with 645 citations. From the table, it can be seen that the concepts of “antibacterial/antimicrobial/anti-infection” and “silver” appeared frequently in titles of articles with high citation rates, and approximately 50% of the article titles were related to anti-infection.

**Table 8:** The references with top 10 high citation.

No.	Title	The first authors	Affiliation	Journal	Publication year	Citations
1	Functionalized nano-MoS2 with peroxidase catalytic and near-infrared photothermal activities for safe and synergetic wound antibacterial applications	Yin, Wenyan	Chinese Academy of Sciences	*ACS Nano*	2016	645
2	Biomedical applications of silver nanoparticles: an up to-date overview	Burdusel, Alexandra-Cristina	Politehnica University of Bucharest	*Nanomaterials*	2018	570
3	Photo-inspired antibacterial activity and wound healing acceleration by hydrogel embedded with Ag/Ag@AgCl/ZnO nanostructures	Mao, Congyang	Hubei University	*ACS Nano*	2017	477
4	Bacterial cellulose in biomedical applications: A review	Picheth, Guilherme Fadel	Federal University of Paraná	*International Journal of Biological Macromolecules*	2017	328
5	In situ synthesis of silver nanoparticles/bacterial cellulose composites for slow-released antimicrobial wound dressing	Wu, Jian	University of Science and Technology Beijing	*Carbohydrate Polymers*	2014	297
6	Conductive adhesive self-healing nanocomposite hydrogel wound dressing for photothermal therapy of infected full-thickness skin wounds	He, Jiahui	Xi’an Jiaotong University	*Chemical Engineering Journal*	2020	262
7	Electrospun nanofibers for wound healing	Liu, Minghuan	Quanzhou Normal University	*Materials Science & Engineering C-Materials for Biological Applications*	2017	259
8	Emerging chitin and chitosan nanofibrous materials for biomedical applications	Ding, Fuyuan	Wuhan University	*Nanoscale*	2014	249
9	Silver nanoparticles: synthesis, medical applications and biosafety	Xu, Li	Central South University	*Theranostics*	2020	240
10	Nanomedicine and advanced technologies for burns: preventing infection and facilitating wound healing	Jahromi, Mirza Ali Mofazzal	Jahrom University of Medical Sciences	*Advanced Drug Delivery Reviews*	2018	231

The top 10 most frequently co-cited articles are presented in Table 9. This aids in comprehending the basis of research and the expertise within a particular area. “*Factors affecting wound healing*,” authored by Guo, S. with 52 citations, has the highest citation frequency in co-citation column. The keywords involved in the co-cited literature are more inclined to macroscopic descriptions of nanomaterials and wound healing. “Anti-infection” still appears frequently, echoing the co-occurring keywords, indicating its importance in nanomaterial research for wound healing.

**Table 9:** The references with top 10 high co-cited frequency.

No.	Title	The first authors	Affiliation	Journal	Publication year	Citations
1	Factors affecting wound healing	Guo, S.	University of Illinois	*Journal of Dental Research*	2010	52
2	Wound repair and regeneration	Gurtner, Geoffrey C.	Stanford University	*Nature*	2008	43
3	Nanotechnology-driven therapeutic interventions in wound healing: potential uses and applications	Hamdan, Suzana	University of Miami	*ACS Central Science*	2017	38
4	Wound healing dressings and drug delivery systems: a review	Boateng, Joshua S.	University of Strathclyde	*Journal of Pharmaceutical Sciences*	2008	38
5	Functionalized nano-MoS2 with peroxidase catalytic and near-infrared photothermal activities for safe and synergetic wound antibacterial applications	Yin, Wenyan	Chinese Academy of Sciences	*ACS Nano*	2016	34
6	Effects of cerium oxide nanoparticles on the growth of keratinocytes, fibroblasts and vascular endothelial cells in cutaneous wound healing	Chigurupati, Srinivasulu	National Institutes of Health (NIH) - USA	*Biomaterials*	2013	33
7	Nanomaterials for wound healing and infection control	Mihai, Mara Madalina	Carol Davila University of Medicine & Pharmacy	*Materials*	2019	31
8	Chronic wound healing: a review of current management and treatments	Han, George	Icahn School of Medicine at Mount Sinai	*Advances in Therapy*	2017	30
9	A review on nanoparticle based treatment for wound healing	Rajendran, Naresh Kumar	University of Johannesburg	*Journal of Drug Delivery Science and Technology*	2018	29
10	Human skin wounds: a major and snowballing threat to public health and the economy	Sen, Chandan K.	The Ohio State University	*Wound Repair and Regeneration*	2009	29

Interestingly, the article “*Functionalized nano-MoS*_*2*_ *with peroxidase catalytic and near-infrared photothermal activities for safe and synergetic wound antibacterial applications*” published in 2016 by Yin, Wenyan, appears in both the top 10 cited list and top 10 co-cited list, indicating it has a high degree of authority as a knowledge repository and is of great significance for the development of nanomaterials in the research area of wound healing.

Figure 5C in CiteSpace presents the timeline of co-cited literature, which is clustered based on keywords, abstract and title. Some important main clusters are used for visual analysis. Cluster #2 appeared relatively early, while Cluster #0 occurred at a comparatively later stage. From the available data, we can deduce that earlier discussions primarily revolved around polymers, whereas in subsequent years, the emphasis shifted to wound dressings. The term “wound dressings” appeared multiple times in various forms in cluster labels, indicating that it has garnered significant attention within this research area.

## 4. Discussion

Over the last 10 years, there has been a consistent rise in the number of research works focusing on nanomaterials in the domain of wound healing. This indicates that nanomaterials have been receiving increasing attention in this field, and researchers’ enthusiasm for them is also growing. This growth trend also indicates that there is still a lot of exploration space in this area of study, moreover, there are numerous matters concerning molecular mechanisms and the prevention and control of diseases that necessitate attention. This subject matter continues to demonstrate its relevance and importance in scientific investigations.

From the analysis of results, it can be seen that the development of studies on nanomaterials in the area of wound healing is not evenly distributed among countries/regions, which could be affected by the importance attached to the medical industry, funding investment, and collaboration between nations. Out of the 65 countries engaged in the study, China is the country with the most publications and citations, followed closely by India, the United States, Iran, South Korea. China’s significant role as a major bridge connecting international scientific research cooperation is evident in its enthusiasm and authority. Likewise, the main affiliations and authors engaged in nanomaterials for wound healing research are mostly from China. In the past decade, the Chinese Academy of Sciences has been the organization with the most extensive number of published works and citations related to this field, and it has extensive and close cooperation with other institutions, which is quite authoritative. The author with the highest H-index (67), Liu, Xiangmei, comes from Hubei University, while the researcher with the most extensive number of published works (12). Zangeneh, Akram, comes from Razi University, and the scholar with the most significant number of citations (792), Grumezescu, Alexandru Mihai, comes from Politehnica University of Bucharest.

Highly cited authors usually also come from organizations with high publication and citation rates. Among these active authors, there exist paired and related cooperation relationships, gradually forming a specific collaboration network of considerable scale. These collaborative networks enhance the exchange and integration of knowledge, promoting the exploration and advancement of scientific study. Additionally, there are institutional collaborations observed, such as a collaboration relationship between 4 authors, Liu, Xiangmei, Wu, Shuilin, Chu, Paul K., and Yang, Xianjin, whose affiliations include Hubei University, Tianjin University, and City University of Hong Kong. The research content of the 4 authors mainly focuses on “silver materials” and “anti-infection.”^[26–29]^ Furthermore, collaborative relationships exist between authors from diverse institutions and countries. Yin, Wenyan, from the Chinese Academy of Sciences, ranks second in terms of citation frequency, and has a collaboration with Chen, Ning from the Ulsan National Institute of Science and Technology in South Korea.^[30]^ Zangeneh, Akram, the author with the most publications, has a collaboration with institutions such as Shanghai Jiao Tong University in China. Although the materials studied by different affiliations are different, they all revolve around “metal materials” and “anti-infection.”^[31–33]^ In fact, the collaborations among authors are relatively scattered, indicating that while nanomaterials receive widespread attention in academia. To foster the advancement and amalgamation of related research, scholars should delve deeper into cooperation and collaboration.

Based on the red cluster in Figure 5A, texts such as “antibacterial,” “infection,” “bacteria,” and “antibiotics” are closely related to “anti-infection.” Skin defects are always accompanied by infection, which has a significant impact on local tissue regeneration and repair, even exacerbating tissue damage and delaying wound healing. Regardless of the cause of full-thickness skin defects, if the wound is not closed, the proliferation of pathogens and the incidence of wound infection are relatively high, and even accompanied by varying degrees of multi-drug resistant bacterial infections.^[34]^ According to studies, the incidence of chronic non-healing wound infections is about 1% to 2% in developed countries, while in developing countries, the incidence of infection is approximately 3%.^[35]^ The progress in nanomaterials and nanotechnology has significantly impacted the field of wound healing, promoting the development of research on wound healing materials. Nanomaterials have great potential in skin defect wound healing and prevention of wound infection due to their adjustable physicochemical properties. Some nanomaterials also have targeting ability to bacterial infection sites. Moreover, due to their large surface area to volume ratio, nanomaterials can significantly increase contact and interaction with microorganisms, activating a wide range of antibacterial mechanisms.^[36]^ In addition, nanomaterials can enhance their antibacterial activity by surface modification or changing their shape. Lately, advancements have been achieved in the study of materials related to photodynamic and phototherapy. Taking black phosphorus as an example, its high bactericidal efficiency through local heating and the production of active oxygen to control infection in wounds is noteworthy.^[37]^ Many studies have shown that local thermal therapy induced by black phosphorus can disrupt the integrity of bacteria, increase blood flow to promote oxygenation and wound healing, and generate reactive oxygen species under light and oxygen, further combating infectious microorganisms to repair wounds.^[38,39]^ Based on the keywords and co-citation analysis shown in Figure 5 and Tables 7 to 9, the term “antibacterial” and other related terms, such as “infection,” “bacteria,” and “antibiotics,” frequently appear and are directly linked to the “anti-infection” cluster in the analysis. This indicates that “anti-infection” is currently a major focus and hot topic in the field of wound healing research involving nanomaterials.

In addition, keyword clustering analysis, timeline chart, high-frequency keyword table, and co-citation graph (Fig. 5A and C, Tables 7–9) indicate that hydrogel wound dressings are also a research hotspot. Research on hydrogel materials has also promoted the development of wound healing. Hydrogel materials can bond damaged tissues, are injectable, have hemostatic and antibacterial properties, serve as drug carriers, and provide a 3-dimensional structure of biomimetic tissue, which are increasingly used as wound dressings,^[40–42]^ consistent with the timeline chart in Figure 5C. A skin substitute made of hydrogel has been developed and can correctly stratify the repaired epidermis after transplantation. It has also developed functional basement membrane and dermal-epidermal junction, displaying nearly normal and functional dermal layer.^[43]^

The table of high-frequency keywords (Table 7) also indicates research on material processes has different focuses, and there is currently no absolute hot direction. This may be a breakthrough point in the research of nanomaterials in the field of wound healing.

Though this research represents the first bibliometric analysis of nanomaterials in the field of wound healing, some common limitations are still present in bibliometric studies. Firstly, balancing completeness and accuracy in search strategies can often be challenging. During the search process, certain relevant articles might be overlooked if the search terms of interest are not present in the search area. Secondly, this study specifically chose Web of Science as the search database due to the compatibility of the visualization software used. While Web of Science is widely recognized as an authoritative academic database, it is important to acknowledge that certain publications might be excluded as they are not part of this database. In addition, because scientific research is continually evolving, and there is a delay in publication, bibliometrics can only analyze the interaction relationships within the literature during a specific timeframe. As the number of citation, publication, and keyword frequency attributes are dynamic and subject to change, this research shows the literature trends related to wound healing and nanomaterials from 2013 to 2022. Future trends in this field still require ongoing research to supplement.

## 5. Conclusions

This research represents the first bibliometric analysis and visualization focusing on nanomaterials in the field of wound healing. The results reveal a clear and intuitive awareness of the distribution of research hotspots and development trends in the field. In the last 10 years, there has been an increasing interest in nanomaterials for wound healing research, with a promising outlook for future studies. The journals *Nanomaterials* and *ACS Nano* have demonstrated considerable activity and influence in nanomaterial research within the wound healing field. China is leading in related research, followed by the United States, and the Chinese Academy of Sciences is the affiliation with the most published studies and citations. The leading researchers in this field are Yin, Wenyan and Grumezescu, Alexandru Mihai. Yin, Wenyan’s article “*Functionalized nano-MoS*_*2*_ *with peroxidase catalytic and near-infrared photothermal activities for safe and synergetic wound antibacterial applications*” published in *ACS Nano* in 2016 is a highly authoritative and relevant academic basis with a strong impact. Finally, infection control has become a major focus and hotspot in this field, closely linking wound healing and nanomaterials. In conclusion, this study uses published articles to extract data and provide an intuitive analysis of the current status and prospects of the field.

## Acknowledgements

We would like to express our gratitude to the individuals and organizations who contributed to the success of this research project. First and foremost, we would like to thank our study participants who generously gave their time and provided valuable insights into the topic. We are also grateful to the healthcare professionals and staff who facilitated our study and made it possible to collect data in a timely manner. Lastly, we would like to express our appreciation to our colleagues and friends who offered their encouragement and feedback along the way.

## Author contributions

**Conceptualization:** Songxia Xia, Liya Yang, Renxian Wang.

**Data curation:** Songxia Xia, Xueshan Bai, Jing-Jun Nie, Renxian Wang.

**Funding acquisition:** Renxian Wang.

**Investigation:** Songxia Xia, Xueshan Bai, Jing-Jun Nie.

**Methodology:** Songxia Xia, Renxian Wang.

**Project administration:** Liya Yang, Renxian Wang.

**Resources:** Li Teng.

**Software:** Songxia Xia.

**Supervision:** Li Teng, Renxian Wang.

**Validation:** Dafu Chen.

**Visualization:** Songxia Xia.

**Writing – original draft:** Songxia Xia, Xueshan Bai.

**Writing – review & editing:** Dafu Chen, Li Teng, Renxian Wang.
